# Vascular Loop Syndrome As the Etiology of Abducens Nerve Palsy: A Case Report

**DOI:** 10.7759/cureus.77483

**Published:** 2025-01-15

**Authors:** Riyaa Rajesh, Rahul Naveen, Warren T Anderson, Ryan Nolan, Rajesh Rangaswamy

**Affiliations:** 1 Department of Biology, University of Southern California, Los Angeles, USA; 2 Department of Radiology, University of Cincinnati College of Medicine, Cincinnati, USA; 3 Department of Interventional Radiology, University of Nevada Reno School of Medicine, Reno, USA; 4 Department of Neurointerventional Radiology, Reno Radiological Associates, Reno, USA

**Keywords:** abducens nerve palsy, diplopia, neuroradiology, vascular loop syndrome, vertebrobasilar artery

## Abstract

Vascular loop syndrome, characterized by arterial loops or kinks causing compression of cranial nerves, can lead to a range of secondary side effects conditional on vascular morphology. This etiology has made standardization of treatment difficult, as confounding factors may take precedence over more interventional treatment. This case seeks to illustrate how vascular loop syndrome, specifically a dolichoectatic vertebrobasilar artery, can lead to left abducens nerve palsy. Here we present an 82-year-old female with reports of diplopia, who was seen previously for sixth nerve palsy. Imaging revealed no acute hemorrhage, restricted diffusion, hydrocephalus, intracranial space-occupying lesion, abnormal volume loss, vasogenic edema, mass effect, or abnormal CSF signal. Magnetic resonance angiography demonstrated an indentation of the left lower brainstem by the tortuous basilar artery, indicating that this vasculature may be abutting the abducens nerve exit zone. We demonstrate a case of vascular loop syndrome as an etiology of abducens nerve palsy and diplopia, raising awareness of these findings on imaging and encouraging the consideration of this etiology in radiological evaluation algorithms.

## Introduction

Abducens, or sixth cranial nerve, palsy is a condition characterized by oculomotor paralysis of the lateral rectus muscle [1]. Etiology for this condition includes impairment or interference of the abducens nerve at any point along its intracranial course [2]. Due to the role of the abducens nerve in somatic control of the lateral rectus muscle, responsible for both ipsilateral eye abduction and partial contralateral eye adduction, damage to this nerve may result in acquired esotropia [1,2]. This misalignment is derived from an inability to abduct the affected eye to counterbalance the antagonistic activity of the medial rectus muscle in controlling eye adduction [2]. Consequently, the affected eye experiences misalignment such that the esotropic deviation is most severe when efforts are made to either abduct the affected eye or fixate at a distance [1,2]. This may manifest as diplopia minimized by strabismus or turning of the head, as well as an inability to abduct the affected eye past the midline [3].

The abducens nerve begins at its nucleus in the dorsal, caudal pons just ventral to the fourth ventricle and exits the brainstem at the junction between the pons and medulla [1]. It then courses both superiorly and anteriorly through the subarachnoid space and over the petrous apex of the temporal bone through a fibrous sheath where it is anchored [1]. The sixth cranial nerve then enters the cavernous sinus, followed by the superior orbital fissure, before innervating the lateral rectus muscle and partially innervating the contralateral medial rectus muscle [1,2]. Due to its long, oblique intracranial course, the abducens nerve is prone to compression or stretching damage that predisposes to abducens nerve palsy [2]. Abducens nerve palsy can be congenital or acquired from trauma, viral or bacterial infections, inflammation, neoplasms and gliomas, aneurysms, variations in intracranial pressure that introduce vascular compromise, microvascular ischemia, fascicular and peripheral lesions, multiple sclerosis, and neurosurgical intervention [3]. Additionally, any etiology that results in aggravation or impairment along the intracranial course of the abducens nerve can manifest as abducens nerve palsy, and its presentation can vary depending on the location at which the cranial nerve is compromised [1]. Onset patterns and associated symptoms can be instrumental in determining etiology. A sudden onset is typically indicative of vascular etiology, a progressive onset suggests a compressive etiology and a subacute onset is characteristic of a demyelinating process [1,2].

In most cases, computed tomography (CT) or magnetic resonance imaging (MRI) reveals some abnormality from which abducens nerve palsy is derived, such as a space-occupying lesion or vascular compromise, that leads to nerve impingement or damage; in other cases where no such abnormality can be found, hypertension or diabetes mellitus is typical [3]. However, in select cases where none of these pathological conditions result in abducens nerve palsy, patients present with this condition secondary to neurovascular compression of the abducens nerve [3]. This disorder, known as vascular loop syndrome, occurs when abnormal movements or pain resulting from the impingement of a cranial nerve by a blood vessel [4,5]. We report an 82-year-old female who presents with diplopia secondary to left abducens nerve palsy and vascular loop syndrome.

## Case presentation

The patient is an 82-year-old female with a history of rheumatoid arthritis, lung cancer, abducens nerve palsy of unknown etiology, and cataracts who presented to an outpatient eye clinic for evaluation of macular degeneration and diplopia that began one month prior. The patient provided verbal and written consent for the publication of this case study. She was subsequently sent to the emergency department to rule out possible transient ischemic attacks. The patient reported right arm numbness and denied headache and abdominal pain. Physical exam was negative for facial droop, slurred speech, and motor weakness.

**Figure 1:**
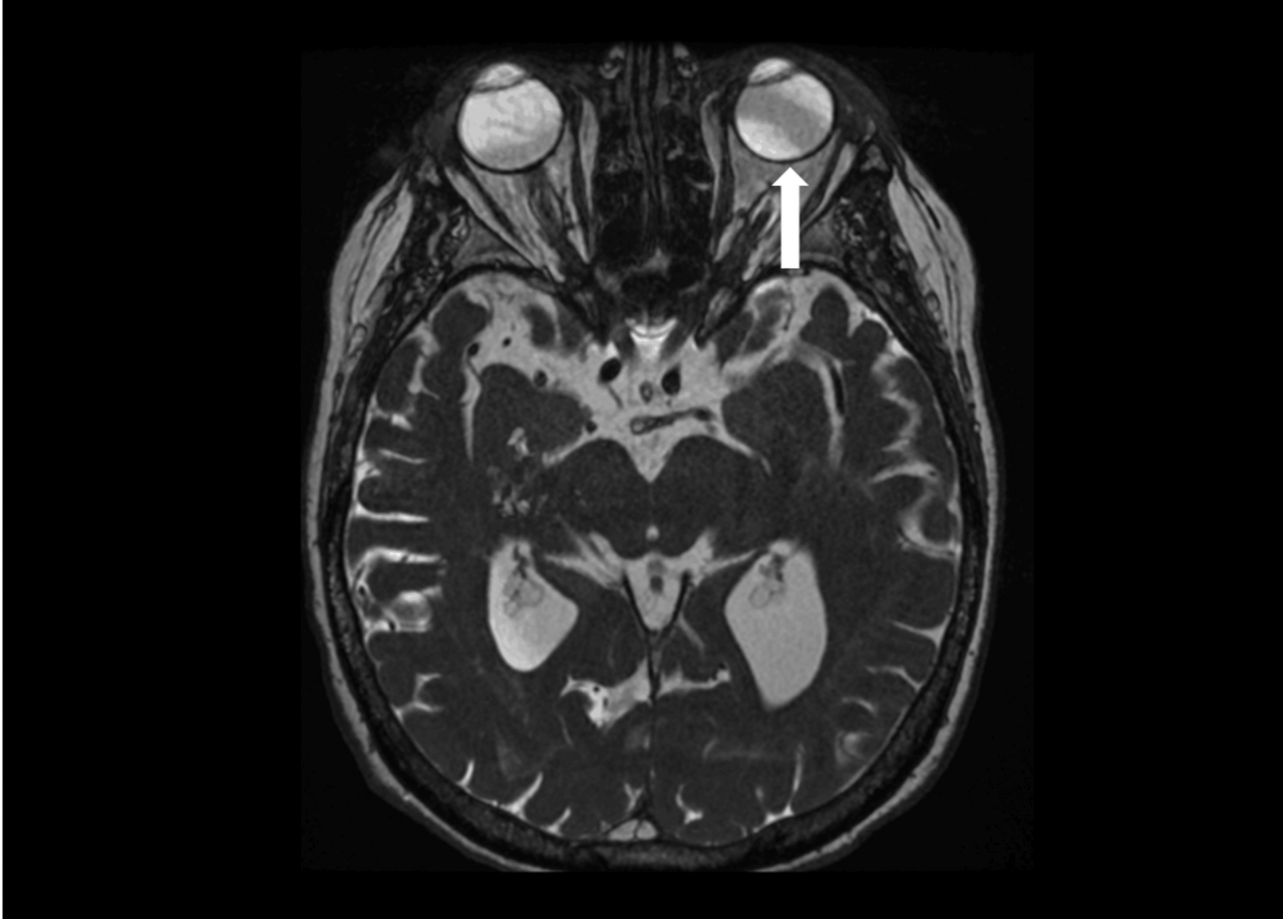
T2-weighted MR demonstrating left eye adduction due to vascular loop syndrome (arrow).

To further evaluate the patient’s diplopia, MRI with and without contrast was ordered. No acute hemorrhage, ischemic lesion, or mass effect was observed on MRI. The left globe was mildly abducted in relation to the right (Figure 1). No significant orbital mass, intraconal or extraconal mass lesions, or abnormal enhancement was observed. Extraocular muscles were normal in caliber and symmetry. The optic nerves, chiasm, and tracts were unremarkable.

Following this finding, the patient decided they wanted to defer microvascular decompression to alleviate her symptoms. The decision to avoid surgery included the risk of complications and advanced age. The patient will be followed by ophthalmology for any changes or worsening of the adduction defect.

**Figure 2:**
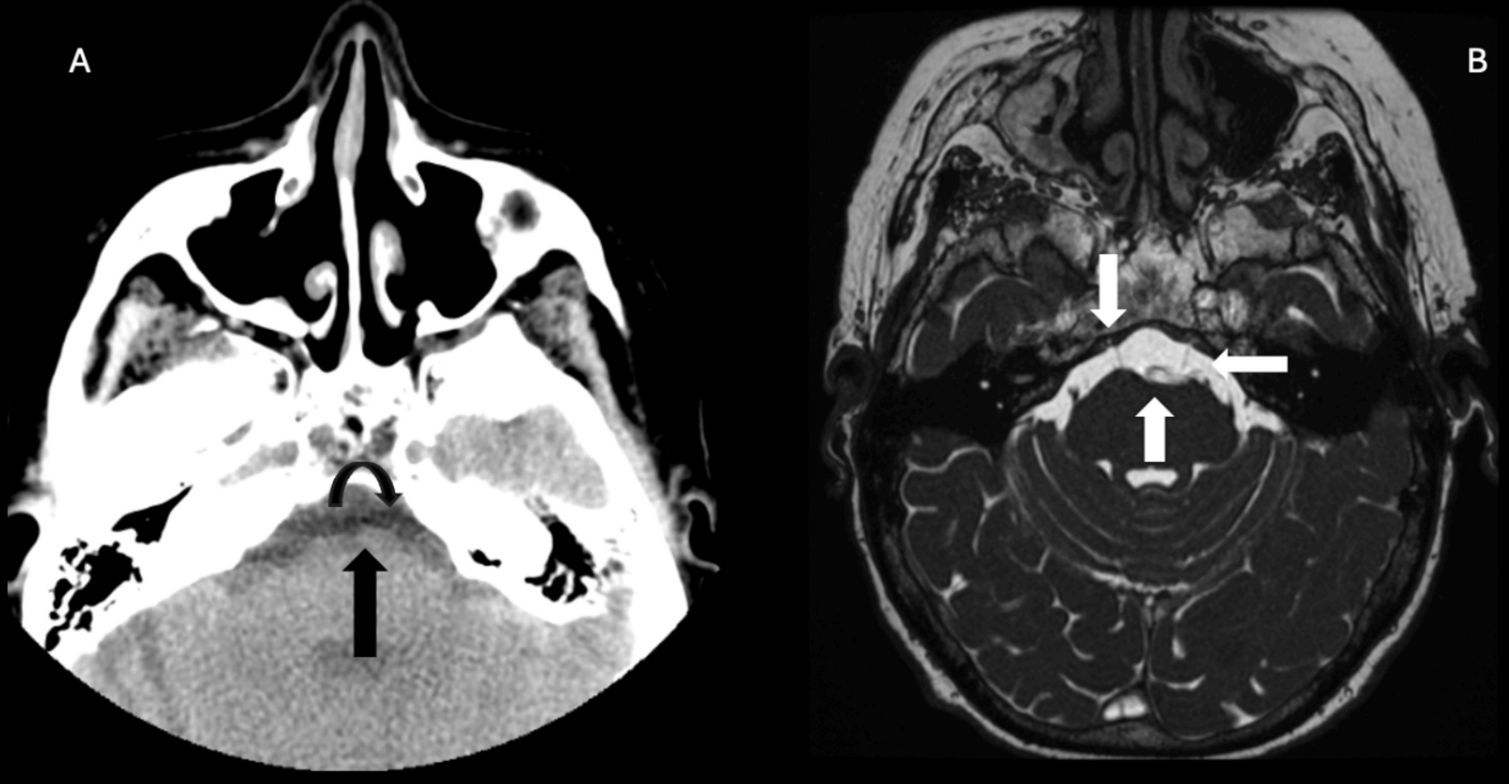
A) Axial CT scan shows the basilar artery intending the left sixth nerve at the exit zone (the vertical arrow shows the basilar artery and the curved arrow shows the left sixth nerve). B) The FIESTA sequences show the basilar artery with indentation of the brain stem at the exit zone (the up arrow shows the basilar artery, the down arrow shows the normal right sixth nerve, and the left arrow shows the compressed left sixth nerve).

Upon brainstem evaluation, an indentation of the left lower brainstem by a tortuous basilar artery, abutting the left abducens nerve exit zone, was noted (Figure 2). Apart from certain areas of gliosis in the brainstem indicative of chronic microvascular ischemia typically seen in old age, no other abnormal lesions were observed along the intracranial course of the left sixth abducens nerve.

## Discussion

In this case report, we present a patient with diplopia who was discovered to have compression of her left abducens nerve due to a tortuous basilar artery. Vascular loop syndrome is characterized by vascular compression of cranial nerves, some of which have been frequently reported in the literature, including the vestibulocochlear and trigeminal nerves, but rarely involve the abducens nerve [6-8]. For example, Teh et al. describe this etiology as a cause of vertigo, while O'Brien et al. describe this as an etiology of tinnitus, chronic headache, and sensorineural hearing loss [6,7]. Furthermore, Aldarwish et al. describe vascular loop syndrome as a possible cause of facial pain due to trigeminal nerve compression [8]. Due to the involvement of various arteries, such as the basilar or anterior inferior cerebellar artery, it is possible for vascular loop syndrome to affect various cranial nerves [7]. Although this etiology is not among common differential diagnoses for a patient with neurological symptoms, it is important to consider this etiology when more likely explanations are failing to provide answers for the patient’s clinical presentation [3].

There are six common etiologies for abducens nerve palsy, the first being brainstem syndrome, which occurs due to a lesion in the posterior fossa [8,9]. The lesion can be inflammatory, compressive, or ischemic and oftentimes includes the fifth, seventh, and eighth cranial nerves. Second, elevated intracranial pressure syndrome results in the displacement of the brainstem, causing the sixth nerve to stretch as it exits the pons [8]. Furthermore, contact between the sixth nerve and the end of the petrous pyramid makes it vulnerable to pathologic processes, otherwise known as petrous syndrome [8]. The cavernous sinus syndrome occurs as the sixth nerve goes through the cavernous sinus. It is associated with the third, fourth, and fifth cranial nerves, the carotid sympathetic plexus, and the internal carotid artery. In this syndrome, dysfunction occurs at either of these structures [3]. The orbital syndrome of the sixth nerve occurs in conjunction with congestion in conjunctival vessels. Oftentimes, it can be hard to distinguish if the third, fourth, or sixth cranial nerves are affected. Last, isolated sixth nerve palsy syndrome occurs in patients who have lateral rectus weakness and no other symptoms indicated in medical history. Risk factors include hypertension and diabetes mellitus, and it is important to rule out the other five syndromes before diagnosing a patient with isolated sixth nerve palsy [3].

Vascular loop syndrome affecting the abducens nerve often manifests as horizontal diplopia, particularly when viewing objects at a distance. This symptom results from ipsilateral lateral rectus muscle weakness, impairing the ability to abduct the affected eye. Some patients adopt compensatory head movements to mitigate diplopia [1,2]. Additional symptoms may include nausea, headache, facial pain, vision loss, and numbness. Variations in symptomatology often depend on underlying etiologies such as intracranial pressure. Elevated intracranial pressure may produce vomiting and eye pain, while cerebrospinal fluid leaks causing low intracranial pressure can mimic high-pressure symptoms, including sixth nerve palsy [1,2].

Microvascular decompression is a potential treatment for patients with vascular loop syndrome due to abducens nerve palsy [10,11]. This procedure involves relieving the vascular compression of the abducens nerve via separation of the vasculature from the exit zone of the nerve [12]. While there has been documented success of this procedure for abducens nerve palsy, this procedure is generally indicated for younger patients who are severely limited by the ocular deficits of this condition [13]. In the case of our patient, she deferred treatment to avoid potential complications. When cases of abducens nerve palsy secondary to vascular loop syndrome are identified, it is necessary to engage in dialogue with the patient to set expectations on the lack of durability and risk of potential complications of surgical treatment for the condition [12,13].

## Conclusions

Ultimately, brainstem imaging identified a tortuous basilar artery compressing the exit of the abducens nerve. Vascular loop syndrome causing compression of the left abducens nerve should be considered as a potential cause of unilateral abducens nerve palsy. This diagnosis is particularly relevant when common etiologies fail to account for the patient’s symptoms. This etiology should be added to the radiologic search pattern for an abducens nerve palsy that is not due to another apparent cause. Prompt recognition and targeted evaluation of this condition are critical for guiding appropriate management, which may vary depending on the patient, age, comorbidities, and goals.
